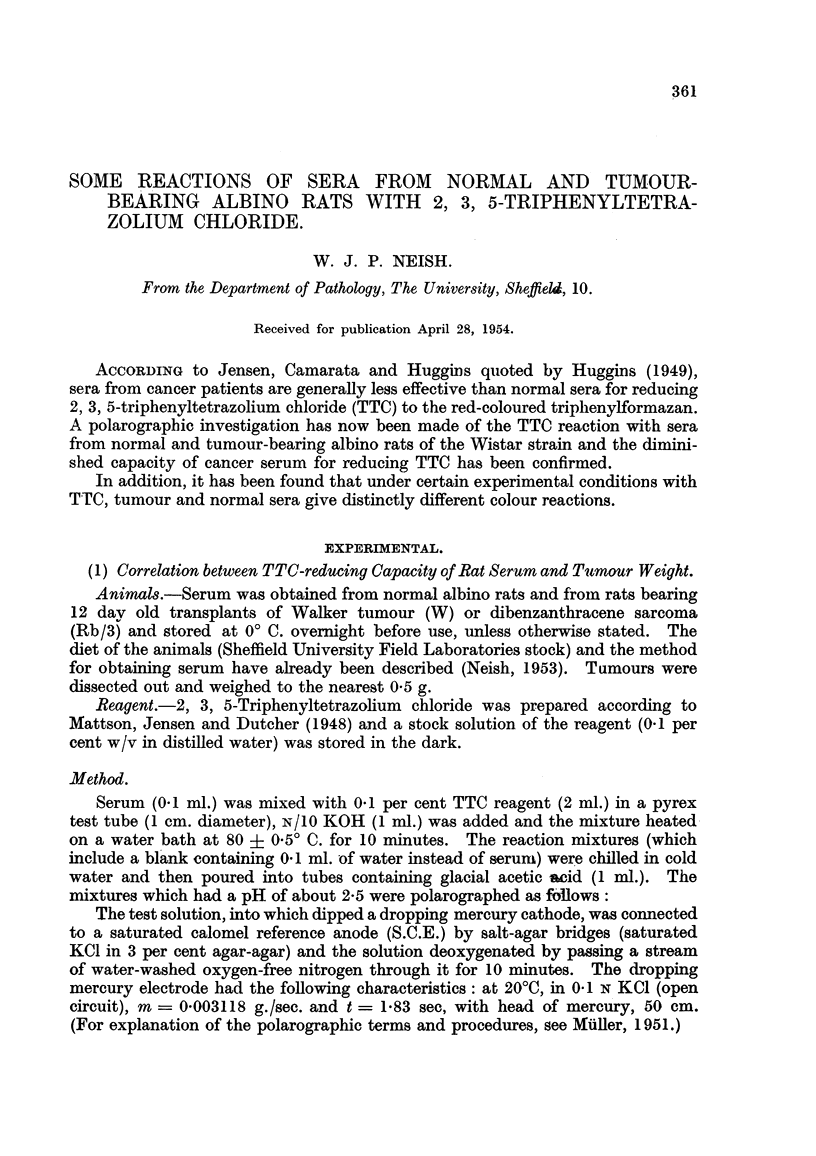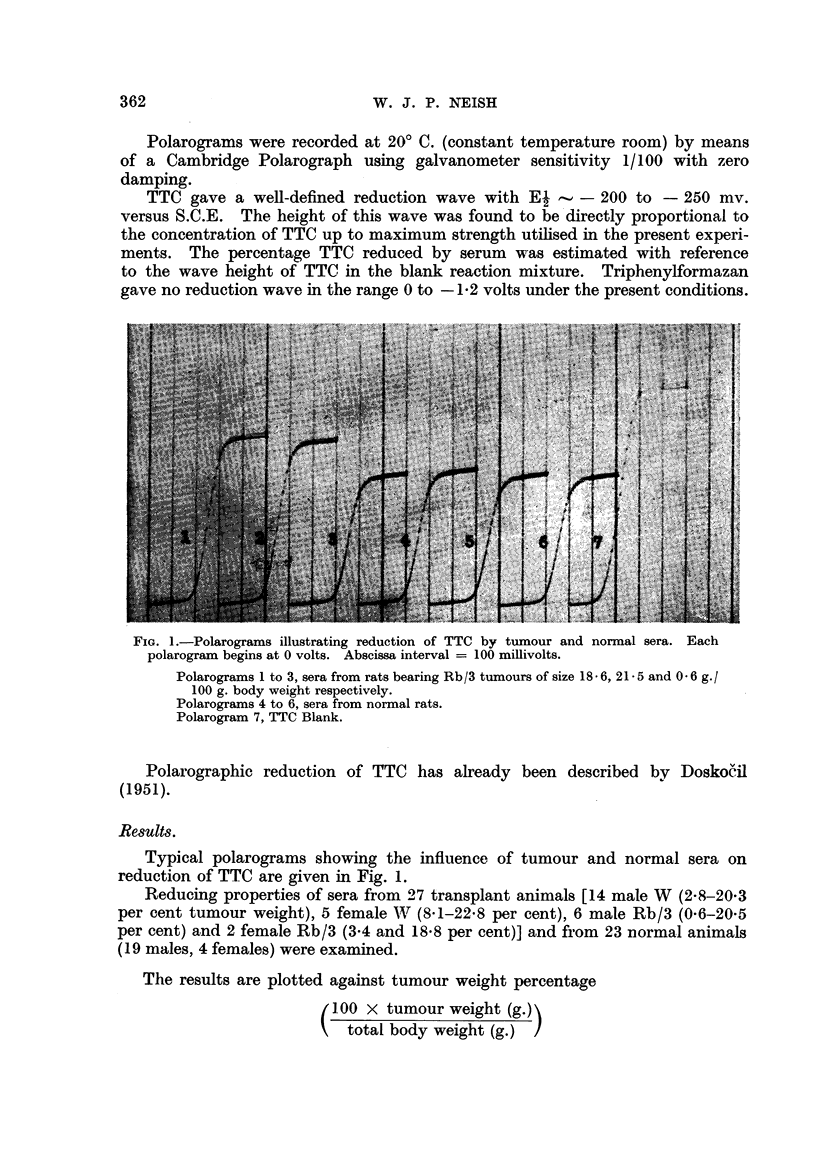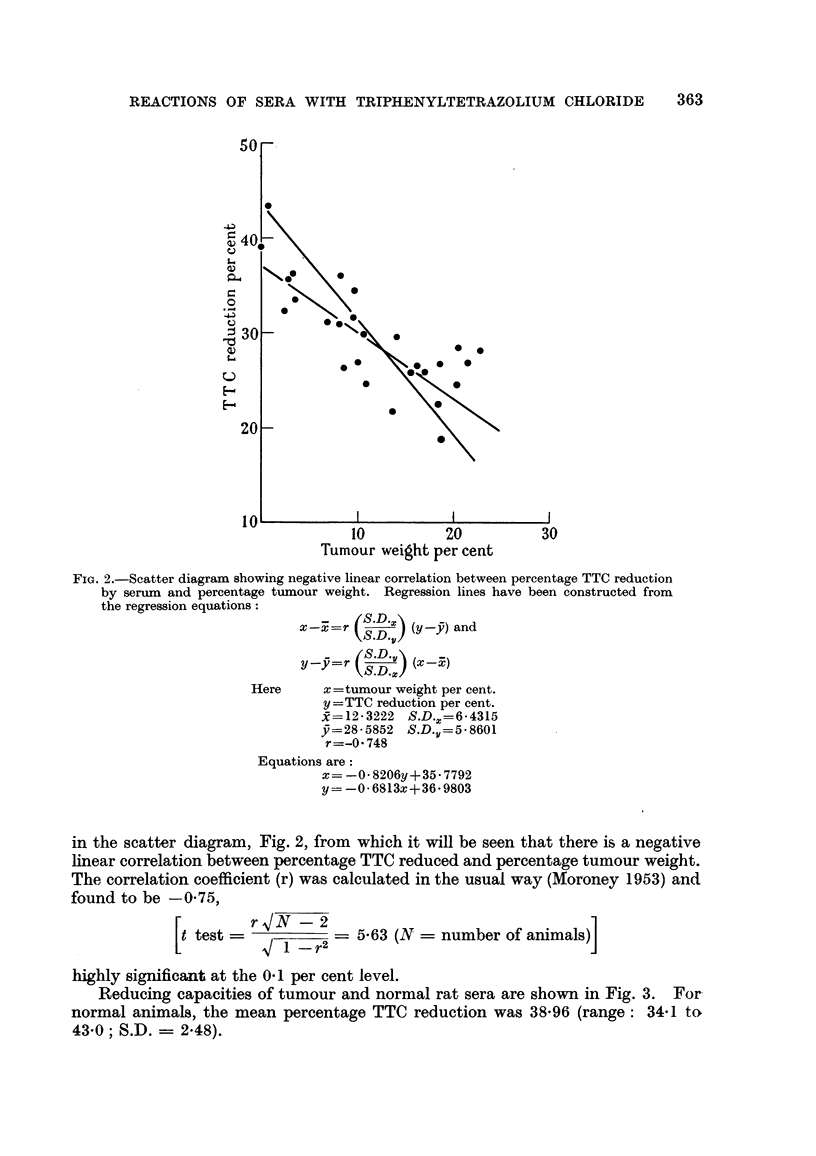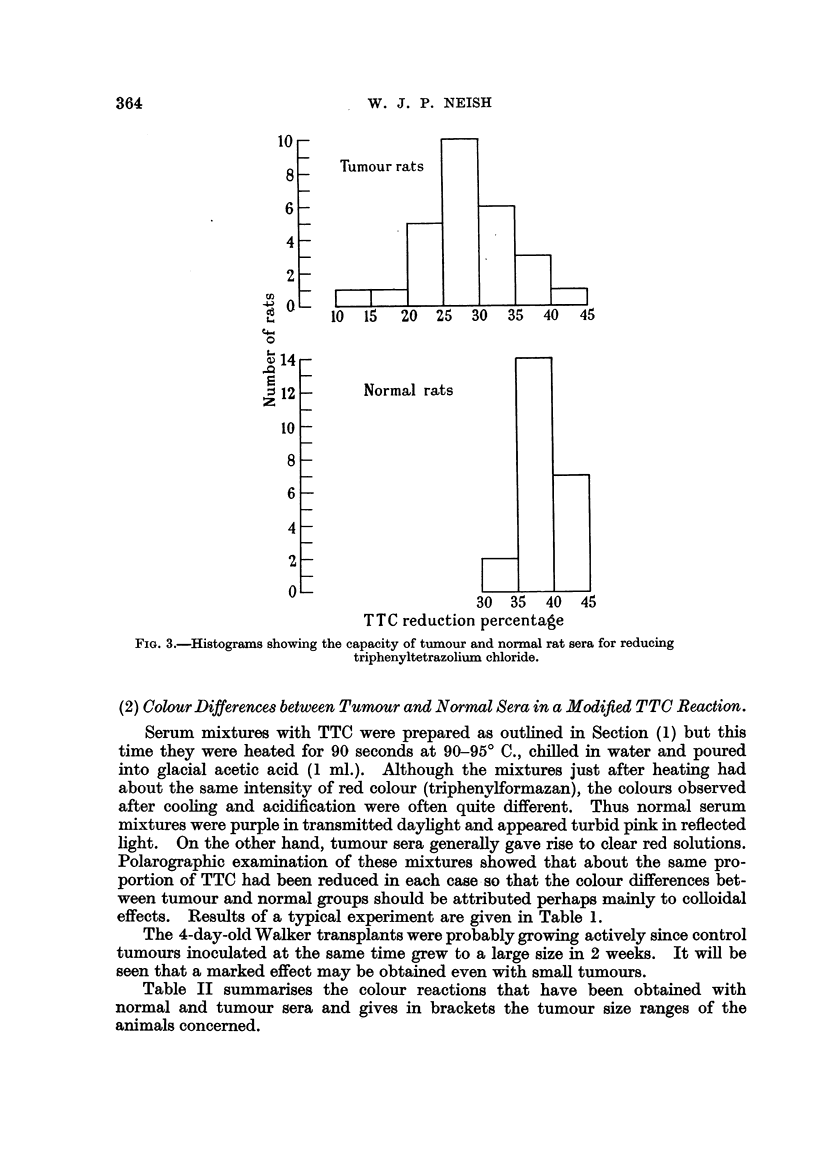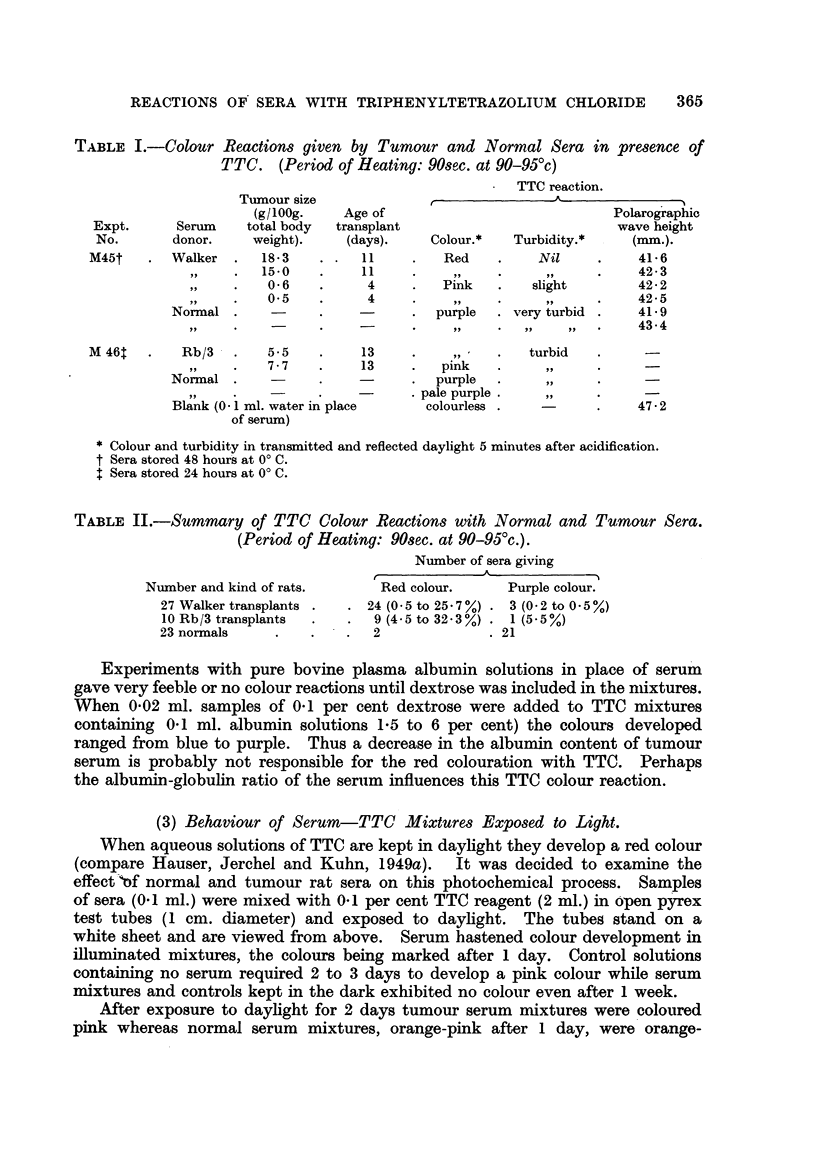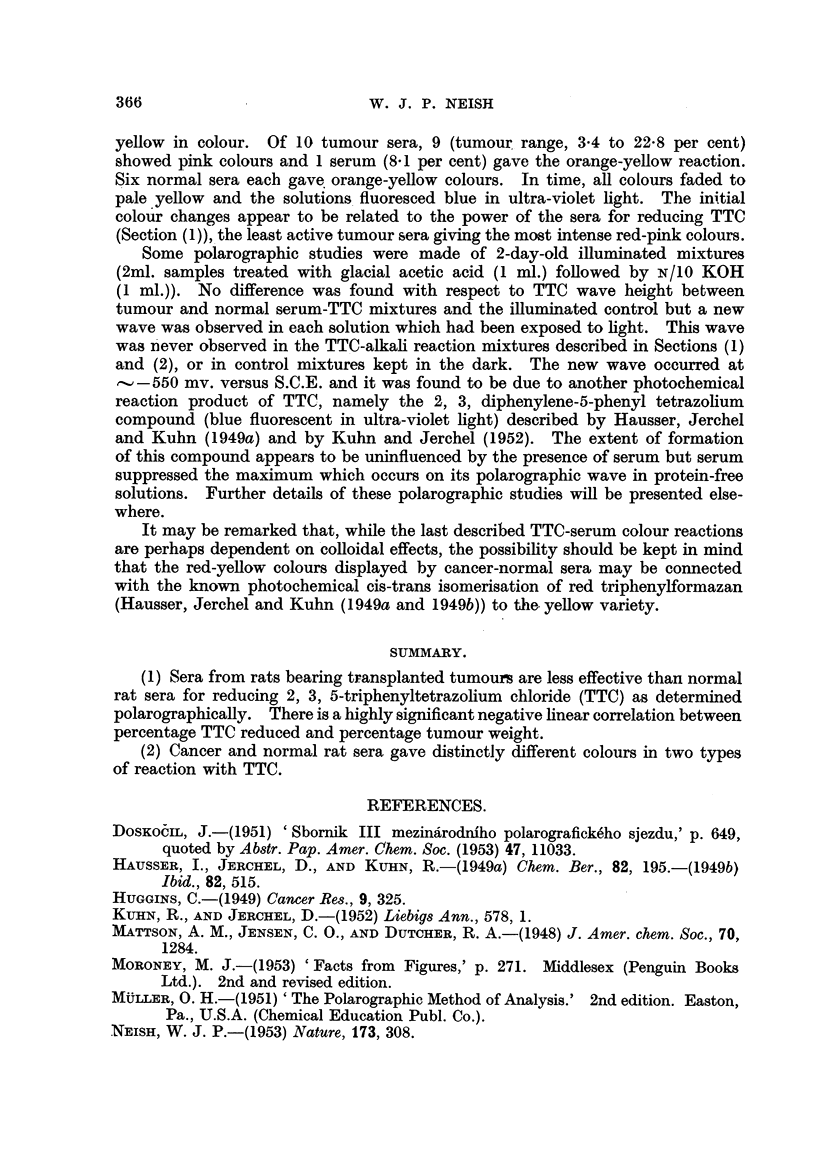# Some Reactions of Sera from Normal and Tumour-bearing Albino Rats with 2, 3, 5-Triphenyltetrazolium Chloride

**DOI:** 10.1038/bjc.1954.37

**Published:** 1954-06

**Authors:** W. J. P. Neish

## Abstract

**Images:**


### SOME REACTIONS OF SERA FROM NORMAL AND TUMOUR-BEARING ALBINO RATS WITH 2, 3, 5-TRIPHENYLTETRAZOLIUM CHLORIDE.

W. J. P. NEISH.

*From the Department of Pathology, The University, Sheffield, 10.*

Received for publication April 28, 1954.

ACCORDING to Jensen, Camarata and Huggins quoted by Huggins (1949), sera from cancer patients are generally less effective than normal sera for reducing 2, 3, 5-triphenyltetrazolium chloride (TTC) to the red-coloured triphenylformazan. A polarographic investigation has now been made of the TTC reaction with sera from normal and tumour-bearing albino rats of the Wistar strain and the diminished capacity of cancer serum for reducing TTC has been confirmed.

In addition, it has been found that under certain experimental conditions with TTC, tumour and normal sera give distinctly different colour reactions.

### EXPERIMENTAL.

### (1) *Correlation between TTC-reducing Capacity of Rat Serum and Tumour Weight.*

*Animals.*—Serum was obtained from normal albino rats and from rats bearing 12 day old transplants of Walker tumour (W) or dibenzanthracene sarcoma (Rb/3) and stored at 0° C. overnight before use, unless otherwise stated. The diet of the animals (Sheffield University Field Laboratories stock) and the method for obtaining serum have already been described (Neish, 1953). Tumours were dissected out and weighed to the nearest 0·5 g.

*Reagent.*—2, 3, 5-Triphenyltetrazolium chloride was prepared according to Mattson, Jensen and Dutcher (1948) and a stock solution of the reagent (0·1 per cent w/v in distilled water) was stored in the dark.

*Method.*

Serum (0·1 ml.) was mixed with 0·1 per cent TTC reagent (2 ml.) in a pyrex test tube (1 cm. diameter), N/10 KOH (1 ml.) was added and the mixture heated on a water bath at 80 ± 0·5° C. for 10 minutes. The reaction mixtures (which include a blank containing 0·1 ml. of water instead of serum) were chilled in cold water and then poured into tubes containing glacial acetic acid (1 ml.). The mixtures which had a pH of about 2·5 were polarographed as follows :

The test solution, into which dipped a dropping mercury cathode, was connected to a saturated calomel reference anode (S.C.E.) by salt-agar bridges (saturated KCl in 3 per cent agar-agar) and the solution deoxygenated by passing a stream of water-washed oxygen-free nitrogen through it for 10 minutes. The dropping mercury electrode had the following characteristics : at 20°C, in 0·1 N KCl (open circuit), $m = 0{\cdot}003118$ g./sec. and $t = 1{\cdot}83$ sec, with head of mercury, 50 cm. (For explanation of the polarographic terms and procedures, see Müller, 1951.)

Polarograms were recorded at 20° C. (constant temperature room) by means of a Cambridge Polarograph using galvanometer sensitivity 1/100 with zero damping.

TTC gave a well-defined reduction wave with $E\frac{1}{2} \sim -200$ to $-250$ mv. versus S.C.E. The height of this wave was found to be directly proportional to the concentration of TTC up to maximum strength utilised in the present experiments. The percentage TTC reduced by serum was estimated with reference to the wave height of TTC in the blank reaction mixture. Triphenylformazan gave no reduction wave in the range 0 to $-1{\cdot}2$ volts under the present conditions.

FIG. 1.—Polarograms illustrating reduction of TTC by tumour and normal sera. Each polarogram begins at 0 volts. Abscissa interval = 100 millivolts.

Polarograms 1 to 3, sera from rats bearing Rb/3 tumours of size 18·6, 21·5 and 0·6 g./100 g. body weight respectively.
Polarograms 4 to 6, sera from normal rats.
Polarogram 7, TTC Blank.

Polarographic reduction of TTC has already been described by Doskočil (1951).

*Results.*

Typical polarograms showing the influence of tumour and normal sera on reduction of TTC are given in Fig. 1.

Reducing properties of sera from 27 transplant animals [14 male W (2·8–20·3 per cent tumour weight), 5 female W (8·1–22·8 per cent), 6 male Rb/3 (0·6–20·5 per cent) and 2 female Rb/3 (3·4 and 18·8 per cent)] and from 23 normal animals (19 males, 4 females) were examined.

The results are plotted against tumour weight percentage

$$\left(\frac{100 \times \text{tumour weight (g.)}}{\text{total body weight (g.)}}\right)$$

FIG. 2.—Scatter diagram showing negative linear correlation between percentage TTC reduction by serum and percentage tumour weight. Regression lines have been constructed from the regression equations :

$$x-\bar{x}=r\left(\frac{S.D._x}{S.D._y}\right)(y-\bar{y}) \text{ and}$$

$$y-\bar{y}=r\left(\frac{S.D._y}{S.D._x}\right)(x-\bar{x})$$

Here $x$ = tumour weight per cent.

$y$ = TTC reduction per cent.

$\bar{x}=12\cdot3222$ $S.D._x=6\cdot4315$

$\bar{y}=28\cdot5852$ $S.D._y=5\cdot8601$

$r=-0\cdot748$

Equations are :
$x=-0\cdot8206y+35\cdot7792$
$y=-0\cdot6813x+36\cdot9803$

in the scatter diagram, Fig. 2, from which it will be seen that there is a negative linear correlation between percentage TTC reduced and percentage tumour weight. The correlation coefficient (r) was calculated in the usual way (Moroney 1953) and found to be —0·75,

$$\left[t \text{ test} = \frac{r\sqrt{N-2}}{\sqrt{1-r^2}} = 5\cdot63 \ (N = \text{number of animals})\right]$$

highly significant at the 0·1 per cent level.

Reducing capacities of tumour and normal rat sera are shown in Fig. 3. For normal animals, the mean percentage TTC reduction was 38·96 (range : 34·1 to 43·0 ; S.D. = 2·48).

FIG. 3.—Histograms showing the capacity of tumour and normal rat sera for reducing triphenyltetrazolium chloride.

(2) *Colour Differences between Tumour and Normal Sera in a Modified TTC Reaction.*

Serum mixtures with TTC were prepared as outlined in Section (1) but this time they were heated for 90 seconds at 90–95° C., chilled in water and poured into glacial acetic acid (1 ml.). Although the mixtures just after heating had about the same intensity of red colour (triphenylformazan), the colours observed after cooling and acidification were often quite different. Thus normal serum mixtures were purple in transmitted daylight and appeared turbid pink in reflected light. On the other hand, tumour sera generally gave rise to clear red solutions. Polarographic examination of these mixtures showed that about the same proportion of TTC had been reduced in each case so that the colour differences between tumour and normal groups should be attributed perhaps mainly to colloidal effects. Results of a typical experiment are given in Table 1.

The 4-day-old Walker transplants were probably growing actively since control tumours inoculated at the same time grew to a large size in 2 weeks. It will be seen that a marked effect may be obtained even with small tumours.

Table II summarises the colour reactions that have been obtained with normal and tumour sera and gives in brackets the tumour size ranges of the animals concerned.

TABLE I.—*Colour Reactions given by Tumour and Normal Sera in presence of TTC. (Period of Heating: 90sec. at 90–95°c)*

| Expt. No. | Serum donor. | Tumour size (g/100g. total body weight). | Age of transplant (days). | TTC reaction. Colour.* | Turbidity.* | Polarographic wave height (mm.). |
|---|---|---|---|---|---|---|
| M45† | Walker | 18·3 | 11 | Red | *Nil* | 41·6 |
| | ,, | 15·0 | 11 | ,, | ,, | 42·3 |
| | ,, | 0·6 | 4 | Pink | slight | 42·2 |
| | ,, | 0·5 | 4 | ,, | ,, | 42·5 |
| | Normal | — | — | purple | very turbid | 41·9 |
| | ,, | — | — | ,, | ,, ,, | 43·4 |
| M 46‡ | Rb/3 | 5·5 | 13 | ,, | turbid | — |
| | ,, | 7·7 | 13 | pink | ,, | — |
| | Normal | — | — | purple | ,, | — |
| | ,, | — | — | pale purple | ,, | — |
| | Blank (0·1 ml. water in place of serum) | | | colourless | — | 47·2 |

* Colour and turbidity in transmitted and reflected daylight 5 minutes after acidification.
† Sera stored 48 hours at 0° C.
‡ Sera stored 24 hours at 0° C.

TABLE II.—*Summary of TTC Colour Reactions with Normal and Tumour Sera. (Period of Heating: 90sec. at 90–95°c.).*

| Number and kind of rats. | Number of sera giving Red colour. | Purple colour. |
|---|---|---|
| 27 Walker transplants | 24 (0·5 to 25·7%) | 3 (0·2 to 0·5%) |
| 10 Rb/3 transplants | 9 (4·5 to 32·3%) | 1 (5·5%) |
| 23 normals | 2 | 21 |

Experiments with pure bovine plasma albumin solutions in place of serum gave very feeble or no colour reactions until dextrose was included in the mixtures. When 0·02 ml. samples of 0·1 per cent dextrose were added to TTC mixtures containing 0·1 ml. albumin solutions 1·5 to 6 per cent) the colours developed ranged from blue to purple. Thus a decrease in the albumin content of tumour serum is probably not responsible for the red colouration with TTC. Perhaps the albumin-globulin ratio of the serum influences this TTC colour reaction.

(3) *Behaviour of Serum—TTC Mixtures Exposed to Light.*

When aqueous solutions of TTC are kept in daylight they develop a red colour (compare Hauser, Jerchel and Kuhn, 1949*a*). It was decided to examine the effect of normal and tumour rat sera on this photochemical process. Samples of sera (0·1 ml.) were mixed with 0·1 per cent TTC reagent (2 ml.) in open pyrex test tubes (1 cm. diameter) and exposed to daylight. The tubes stand on a white sheet and are viewed from above. Serum hastened colour development in illuminated mixtures, the colours being marked after 1 day. Control solutions containing no serum required 2 to 3 days to develop a pink colour while serum mixtures and controls kept in the dark exhibited no colour even after 1 week.

After exposure to daylight for 2 days tumour serum mixtures were coloured pink whereas normal serum mixtures, orange-pink after 1 day, were orangeyellow in colour. Of 10 tumour sera, 9 (tumour range, 3·4 to 22·8 per cent) showed pink colours and 1 serum (8·1 per cent) gave the orange-yellow reaction. Six normal sera each gave orange-yellow colours. In time, all colours faded to pale yellow and the solutions fluoresced blue in ultra-violet light. The initial colour changes appear to be related to the power of the sera for reducing TTC (Section (1)), the least active tumour sera giving the most intense red-pink colours.

Some polarographic studies were made of 2-day-old illuminated mixtures (2ml. samples treated with glacial acetic acid (1 ml.) followed by N/10 KOH (1 ml.)). No difference was found with respect to TTC wave height between tumour and normal serum-TTC mixtures and the illuminated control but a new wave was observed in each solution which had been exposed to light. This wave was never observed in the TTC-alkali reaction mixtures described in Sections (1) and (2), or in control mixtures kept in the dark. The new wave occurred at $\sim -550$ mv. versus S.C.E. and it was found to be due to another photochemical reaction product of TTC, namely the 2, 3, diphenylene-5-phenyl tetrazolium compound (blue fluorescent in ultra-violet light) described by Hausser, Jerchel and Kuhn (1949*a*) and by Kuhn and Jerchel (1952). The extent of formation of this compound appears to be uninfluenced by the presence of serum but serum suppressed the maximum which occurs on its polarographic wave in protein-free solutions. Further details of these polarographic studies will be presented elsewhere.

It may be remarked that, while the last described TTC-serum colour reactions are perhaps dependent on colloidal effects, the possibility should be kept in mind that the red-yellow colours displayed by cancer-normal sera may be connected with the known photochemical cis-trans isomerisation of red triphenylformazan (Hausser, Jerchel and Kuhn (1949*a* and 1949*b*)) to the yellow variety.

### SUMMARY.

(1) Sera from rats bearing transplanted tumours are less effective than normal rat sera for reducing 2, 3, 5-triphenyltetrazolium chloride (TTC) as determined polarographically. There is a highly significant negative linear correlation between percentage TTC reduced and percentage tumour weight.

(2) Cancer and normal rat sera gave distinctly different colours in two types of reaction with TTC.

### REFERENCES.

Doskočil, J.—(1951) 'Sbornik III mezinárodního polarografického sjezdu,' p. 649, quoted by *Abstr. Pap. Amer. Chem. Soc.* (1953) **47**, 11033.

Hausser, I., Jerchel, D., and Kuhn, R.—(1949*a*) *Chem. Ber.*, **82**, 195.—(1949*b*) *Ibid.*, **82**, 515.

Huggins, C.—(1949) *Cancer Res.*, **9**, 325.

Kuhn, R., and Jerchel, D.—(1952) *Liebigs Ann.*, 578, 1.

Mattson, A. M., Jensen, C. O., and Dutcher, R. A.—(1948) *J. Amer. chem. Soc.*, **70**, 1284.

Moroney, M. J.—(1953) 'Facts from Figures,' p. 271. Middlesex (Penguin Books Ltd.). 2nd and revised edition.

Müller, O. H.—(1951) 'The Polarographic Method of Analysis.' 2nd edition. Easton, Pa., U.S.A. (Chemical Education Publ. Co.).

Neish, W. J. P.—(1953) *Nature*, **173**, 308.